# Development of a PET/CT molecular radiomics-clinical model to predict thoracic lymph node metastasis of invasive lung adenocarcinoma ≤ 3 cm in diameter

**DOI:** 10.1186/s13550-022-00895-x

**Published:** 2022-04-21

**Authors:** Cheng Chang, Maomei Ruan, Bei Lei, Hong Yu, Wenlu Zhao, Yaqiong Ge, Shaofeng Duan, Wenjing Teng, Qianfu Wu, Xiaohua Qian, Lihua Wang, Hui Yan, Ciyi Liu, Liu Liu, Jian Feng, Wenhui Xie

**Affiliations:** 1 Department of Nuclear Medicine, Shanghai Chest Hospital, Shanghai Jiao Tong University, No. 241 West Huaihai Road, Shanghai, 200030 China; 2 Clinical and Translational Center in Shanghai Chest Hospital, Shanghai Key Laboratory for Molecular Imaging, Shanghai University of Medicine and Health Sciences, Shanghai, China; 3 Department of Radiology, Shanghai Chest Hospital, Shanghai Jiao Tong University, Shanghai, China; 4 Department of Radiology, Second Affiliated Hospital of Soochow University, Suzhou, Jiangsu China; 5 GE Healthcare China, Pudong New Town, Shanghai, China; 6 Shanghai Municipal Hospital of Traditional Chinese Medicine, Shanghai University of Traditional Chinese Medicine, Shanghai, China; 7 Institute for Medical Imaging Technology, School of Biomedical Engineering, Shanghai Jiao Tong University, Shanghai, China; 8 Department of Thoracic Surgery, Shanghai Chest Hospital, Shanghai Jiao Tong University, Shanghai, China

**Keywords:** CT, PET/CT, Radiomics, Lymph node metastasis, Lung adenocarcinoma

## Abstract

**Background:**

To investigate the value of ^18^F-FDG PET/CT molecular radiomics combined with a clinical model in predicting thoracic lymph node metastasis (LNM) in invasive lung adenocarcinoma (≤ 3 cm).

**Methods:**

A total of 528 lung adenocarcinoma patients were enrolled in this retrospective study. Five models were developed for the prediction of thoracic LNM, including PET radiomics, CT radiomics, PET/CT radiomics, clinical and integrated PET/CT radiomics-clinical models. Ten PET/CT radiomics features and two clinical characteristics were selected for the construction of the integrated PET/CT radiomics-clinical model. The predictive performance of all models was examined by receiver operating characteristic (ROC) curve analysis, and clinical utility was validated by nomogram analysis and decision curve analysis (DCA).

**Results:**

According to ROC curve analysis, the integrated PET/CT molecular radiomics-clinical model outperformed the clinical model and the three other radiomics models, and the area under the curve (AUC) values of the integrated model were 0.95 (95% CI: 0.93–0.97) in the training group and 0.94 (95% CI: 0.89–0.97) in the test group. The nomogram analysis and DCA confirmed the clinical application value of this integrated model in predicting thoracic LNM.

**Conclusions:**

The integrated PET/CT molecular radiomics-clinical model proposed in this study can ensure a higher level of accuracy in predicting the thoracic LNM of clinical invasive lung adenocarcinoma (≤ 3 cm) compared with the radiomics model or clinical model alone.

**Supplementary Information:**

The online version contains supplementary material available at 10.1186/s13550-022-00895-x.

## Background

Lung adenocarcinoma accounts for over 30% of all lung cancers. Furthermore, approximately 20% of patients with invasive lung adenocarcinoma (≤ 3 cm) already have thoracic lymph node metastasis(LNM)at the time of diagnosis [1, 2]. Identifying the presence of thoracic LNM before surgery can indicate the necessity of intraoperative mediastinal lymph node dissection and subsequent radical resection in some invasive lung adenocarcinomas (≤ 3 cm) [2]. In addition, determining LNM status in advance is also important for the selection of the target range of radiotherapy [3]. Unfortunately, conventional approaches to thoracic LNM detection, thoracoscopy and transbronchial biopsies can lead to complications, including haemorrhage, infection and pneumothorax, which increases the cost of treatment despite high accuracy [4]. In addition, positron emission tomography/computed tomography (PET/CT), considered the most accurate thoracic LNM staging method, is subject to low diagnostic sensitivity due to several factors, such as lymph node hypertrophy, false positives caused by infection and inflammation and limited spatial resolution [5–9]. For example, Liu et al*.* showed that the sensitivity and specificity of PET/CT in the preoperative diagnosis of mediastinal LNM in non-small-cell lung cancer (NSCLC) patients were 65% and 96.8%, respectively [8]. On the one hand, the micrometastasis of certain lymph nodes with a diameter smaller than the spatial resolution range of PET/CT may cause false negatives [10]. On the other hand, long-term smoking and lung infections can lead to false positives [11]. In addition, PET/CT imaging reading is dependent on visual assessment and semiautomated measurements, which in turn rely on different interpretations by different observers. The accuracy of PET/CT in predicting the incidence of occult LNM in NSCLC is only approximately 14–19% [12, 13].

As a supplement to the abovementioned approaches, radiomics can help improve the diagnostic efficiency of lung cancer LNM [14]. Coroller et al*.* reported 35 CT radiomics features of lung adenocarcinoma for predicting distant metastasis [15]. Yang et al*.* demonstrated the value of CT radiomics in predicting lung adenocarcinoma LNM [16]. Cong et al*.* used the random forest method to establish models to predict the LNM of lung adenocarcinoma [17]. Although the CT-based evaluation of radiomics features has been shown to be a promising predictor of lung adenocarcinoma LNM, PET/CT molecular radiomics-clinical models, including PET/CT molecular radiomics features and clinical factors, have not been investigated for their potential in predicting the thoracic LNM of lung adenocarcinoma**.** To address the literature gap, we conducted a comparative study of five models, including integrated PET/CT molecular radiomics-clinical, PET/CT radiomics, PET radiomics, CT radiomics, and clinical models, for predicting the thoracic LNM of lung adenocarcinoma. Among them, for PET/CT radiomics, the primary lesions on PET and CT images were delineated before extracting and analysing radiomics features [18]. The current study explored the diagnostic efficacy of PET/CT, CT, and PET radiomics models in predicting the thoracic LNM of lung adenocarcinoma by analysing the features of PET/CT images of lung adenocarcinoma patients. The study aimed to develop an integrated PET/CT molecular radiomics-clinical model for predicting the thoracic LNM of invasive lung adenocarcinoma (≤ 3 cm).

## Methods

### Patient selection and pathological evaluation

A retrospective analysis of 802 patients with lung adenocarcinoma (diameter ≤ 3 cm) was performed in Shanghai Chest Hospital from February 2016 to January 2021. The inclusion and exclusion criteria are illustrated in Additional file 1: Figure S1. A total of 528 patients with invasive lung adenocarcinoma were enrolled, including 379 patients with lung adenocarcinoma without thoracic LNM, accounting for 71.78%, and 149 patients with lung adenocarcinoma with thoracic LNM, accounting for 28.22%. Thoracic LNM was defined as lung cancer with N1 or N2 LNM. All patients underwent surgeries after diagnosis, including N1 and N2 resection. Two pathologists evaluated the tumour histology of these patients by following the 2015 WHO classification of lung adenocarcinoma. Lymph node staging was defined according to the eighth version of the TNM staging method. The study protocol was approved by the institutional ethics review committee of Shanghai Chest Hospital. Informed consent was not required because of the retrospective nature of the study. All the patient data were anonymized in this paper.

### Clinical information of the selected lung adenocarcinoma patients

The clinical information of the patients included age, sex, smoking history, and the tumour marker carcinoembryonic antigen (CEA). The CT features included lung tumour location, lobulation sign, burr sign, pleural traction, and solid component size. The PET parameter included the maximum standardized uptake value (SUVmax). The clinical information of the enrolled patients is summarized in Table 1. The size of the solid component of pulmonary nodules, including mixed ground-glass nodules, refers to the average value of the longest cross-section length and the vertical diameter length of the solid component on the pulmonary window [19].

**Table 1:** Clinical features of 528 patients enrolled in this study

Clinical characteristics	Training (*n* = 371)			Testing (*n* = 157)		
	Lymph node (−)	Lymph node (+)	*p* value	Lymph node (−)	Lymph node (+)	*p* value
Age, year (median;IQR)	62; 55 ~ 67	62; 55 ~ 68	0.62	63; 56 ~ 69	60; 50 ~ 70	0.166
*Gender*						
Male	103	46	0.369	48	23	0.555
Female	163	59		65	21	
*Smoke*						
Yes	84	36	0.617	38	15	0.958
No	182	69		75	29	
*Location*						
Upper lobe, right	109	30	0.024	48	12	0.101
Middle lobe, right	46	18		21	10	
Lower lobe, right	13	8		5	1	
Upper lobe, left	60	29		26	15	
Lower lobe, left	38	20		13	6	
CEA, ng/ml (median; IQR)	2.32; 1.53 ~ 3.93	3.67; 2.11 ~ 8.50	0.273	2.21; 1.53 ~ 3.52	3.59; 2.01 ~ 6.27	0.251
*Lobulation*						
(+)	260	103	0.836	112	44	0.279
(−)	6	2		1	0	
*Burr*						
(+)	249	102	0.176	112	44	0.688
(−)	17	3		1	0	
*Pleural traction*						
(+)	45	53	< 0.001	15	18	< 0.001
(−)	221	52		98	26	
Solid components, cm (median; IQR)	0.85; 0.50 ~ 1.3	2.05; 1.65 ~ 2.45	< 0.001	0.95; 0.55 ~ 1.4	2.1; 1.65 ~ 2.49	< 0.001
SUVmax (median;IQR)	3.68; 2.1 ~ 7.45	11.11; 8.92 ~ 15.31	0.145	3.57; 2.08 ~ 6.99	10.25; 7.1 ~ 13.36	0.279

### PET/CT scan procedures

All patients were examined under the same scanning conditions on the same device (Siemens Biograph MCT-S PET/CT). A 64-slice spiral CT was used. ^18^F-fluorodeoxyglucose (^18^F-FDG) was provided by Shanghai Atom Kexing Pharmaceuticals Co., Ltd. All patients had withheld from eating and drinking for more than 6 h before the PET/CT procedure, thus keeping the blood glucose level below 150 mg/dL. All patients were injected with ^18^F-FDG at 5 MBq/kg ± 10% of body weight and then rested for 60 min. The PET scan was divided into 5 or 6 beds, and each bed was checked for approximately 2 min. The CT data were used to attenuate corrected PET images, and Truex + TOF was used to reconstruct PET images. The PET and CT scan thickness of all patients was 5 mm. The matrix size of all PET reconstructions was 200 × 200, and the anisotropic voxel was 4.07 × 4.07 × 3.0 mm^3^. After regular PET and CT scans, a 1 mm breath-hold lung CT scan was added. CT was reconstructed by a conventional algorithm, while PET was reconstructed by an iterative method.

### Lesion segmentation

**Fig. 1:**
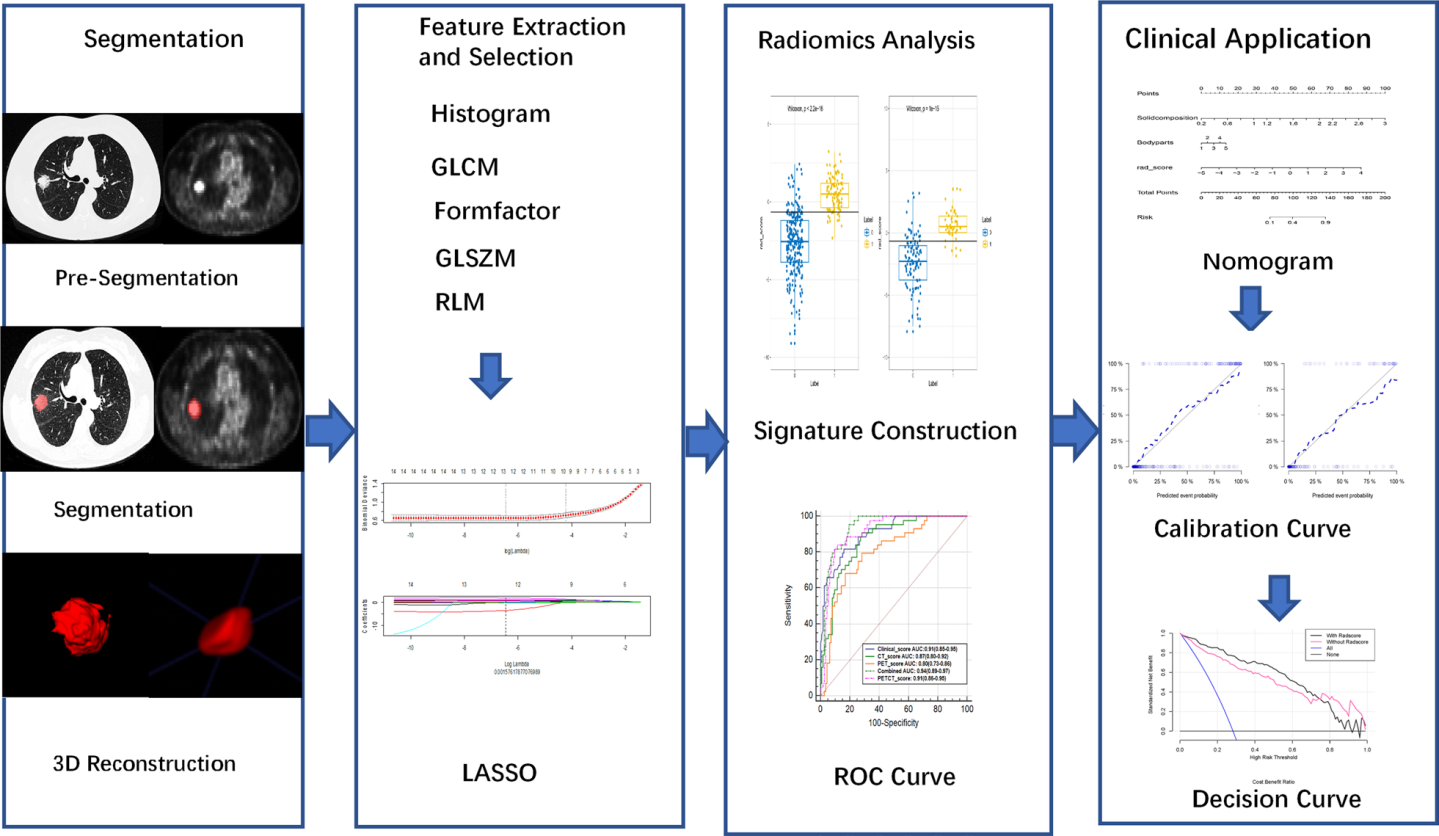
Workflow for developing a radiomics model based on PET/CT images to predict the thoracic LNM of lung adenocarcinoma. GLCM, gray level co-occurrence matrix; GLSZM, grey level size zone matrix; RLM, run length matrix; mRMR, maximum relevance minimum redundancy; LASSO, least absolute shrinkage and selection operator; ROC, receiver operating characteristic

The 5-mm slice thickness PET images and 1-mm slice thickness CT images of all patients were exported from the PACS workstation in DICOM format and then imported into ITK-SNAP software (version 3.8.0-beta, www.itksnap.org) to outline lung lesions in 3D mode. The entire delineation procedure was performed by two radiologists with over 10 years of work experience, and neither of the radiologists was informed of the patients’ pathological results. For CT image delineation, the lesion was observed on the lung window (window width 1600 HU, window level -600 HU). The two radiologists delineated the primary tumour on PET images using a 40% SUVmax threshold to characterize the volume of interest (VOI) [20–24]. To avoid including the physiologic uptake in the VOI, a combined CT and PET scan reading was performed [22, 24]. An example of VOI delineation is shown in Fig. 1.

### Image preprocessing

The original 5 mm PET, 1 mm breath-holding thin-layer CT (DICOM format), and outlined VOI of each lung tumour were imported into IBSI-compatible Artificial Intelligence Kit software (AK analysis kit, GE healthcare, 3.2.2) for image preprocessing [23–27]. The μ ± 3σ method was used to remove data with a brightness greater than 3σ to normalize image brightness [23, 24, 28]. The images were resampled to 1 × 1 × 1 mm^3^ by using linear interpolation to improve the image resolution. The preprocessed images were then imported into ITK-SNAP to delineate the VOI.

### Feature extraction and selection

The inter- and intraclass correlation coefficients (ICCs) were evaluated, in which 50 cases were randomly selected from the enrolled study cases. Two observers (Observers A and B) with more than 10 years of working experience in PET and CT applications delineated the VOIs. Observer A delineated the VOIs of CT and PET images twice at an interval of 4 weeks, and the intraobserver correlation coefficients of the extracted features were evaluated between the two delineations of Observer A. Observer B delineated the VOIs independently once, and the interobserver correlation coefficients between the radiomics features extracted by Observers A (the first delineation) and B were evaluated. ICC > 0.75 indicates good agreement. Observer A then finished the remaining delineation work. Based on the VOIs of lung tumours outlined by Observer A on CT and PET images, 402 radiomics features were extracted from every image by using AK software, including 42 histograms features, 154 grey level co-occurrence matrix (GLCM) features, 15 formfactor features describing the shape of the VOI, 180 run length matrix (RLM) features and 11 grey level size zone matrix (GLSZM) features. The bin width was set to 25 during feature extraction.

### Model construction and validation

The patients were randomly assigned into training (371 patients) and test groups (157 patients) at a ratio of 7 to 3 using a stratified sampling method to ensure the balance of positive and negative samples in both groups [20, 23, 28]. Bootstrapping was used to split the data into training and validation groups. For the bootstrap samples, simple random sampling was used. To improve the representativeness of the minority group in the training group, the synthetic minority oversampling technique (SMOTE) was used to generate samples of the minority group from the joint weighting of optimal features. In the training group, the maximum relevance minimum redundancy (mRMR) and least absolute shrinkage and selection operator (LASSO) methods were applied to select the most valuable radiomics features (ICC > 0.75) for predicting lung adenocarcinoma LNM. Three multivariate logistic regression models based on PET/CT, CT, and PET were then established in the training group.

The radiomic score of each patient was calculated based on the combination of the retained features weighted by their LASSO logistic regression coefficients (Additional file 6: Methods). The area under the curve (AUC) was used to evaluate the diagnostic efficacy of the three radiomics models in predicting the thoracic LNM of lung adenocarcinoma. The efficacy of predicting the thoracic LNM of lung adenocarcinoma was evaluated in the test group. The DeLong test was employed to compare the performance of the three different models based on PET/CT, CT, and PET to determine the most powerful predictive model. To verify the reliability of the model, a cross-validation test was performed 100 times. The workflow of radiomics analysis is shown in Fig. 1.

### Construction of the radiomics nomogram

The clinical factors (*p* < 0.1) were analysed using univariate logistic regression to identify whether the features were discriminative (*p* < 0.05). Then, multivariate logistic regression was applied to these discriminative clinical features to construct a clinical model, and the clinical features, as well as the radiomics score, were integrated to establish a predictive nomogram. Moreover, the variance inflation factor (VIF) was used for collinearity analysis, and factors with VIF > 10 were eliminated. All the models were constructed in the training group and then validated in the test group.

### Statistical analysis

In this study, the programming language R (software version 3.5.1) was used for statistical analysis. For clinical data, the chi-square test was applied to features with a normal distribution, which were presented as the mean ± SD, while the Wilcoxon test was applied to features with a nonnormal distribution, which were presented as the median (lower and upper quartiles). In this study, the *ModelGood* package of R was used to construct the calibration curve. Decision curve analysis (DCA) was used to evaluate the clinical value of the PET/CT molecular radiomics-clinical model in predicting lung adenocarcinoma LNM in the test group.

## Results

### Radiomics feature extraction and selection

For the radiomics features extracted twice by Observer A, the intra-ICC ranges in the CT group and PET group were 0.06–1 and 0.32–1, respectively. For the features extracted by Observer A (for the first time) and Observer B, the inter-ICC ranges in the CT groups and PET group were 0.15–1 and 0.3–1, respectively, and the features with ICC > 0.75 in both the intragroup and intergroup comparisons were retained for further analysis (Additional file 5: Table S1). The PET/CT, CT, and PET data sets were further analysed by the mRMR algorithm and LASSO regression model. After feature extraction and selection, 10 PET/CT (6 CT and 4 PET), 12 CT, and 10 PET radiomics features were retained. These features and their corresponding coefficients are shown in Additional file 2: Figures S2–4.

### Evaluation of the performance of the three radiomics models

**Fig. 2:**
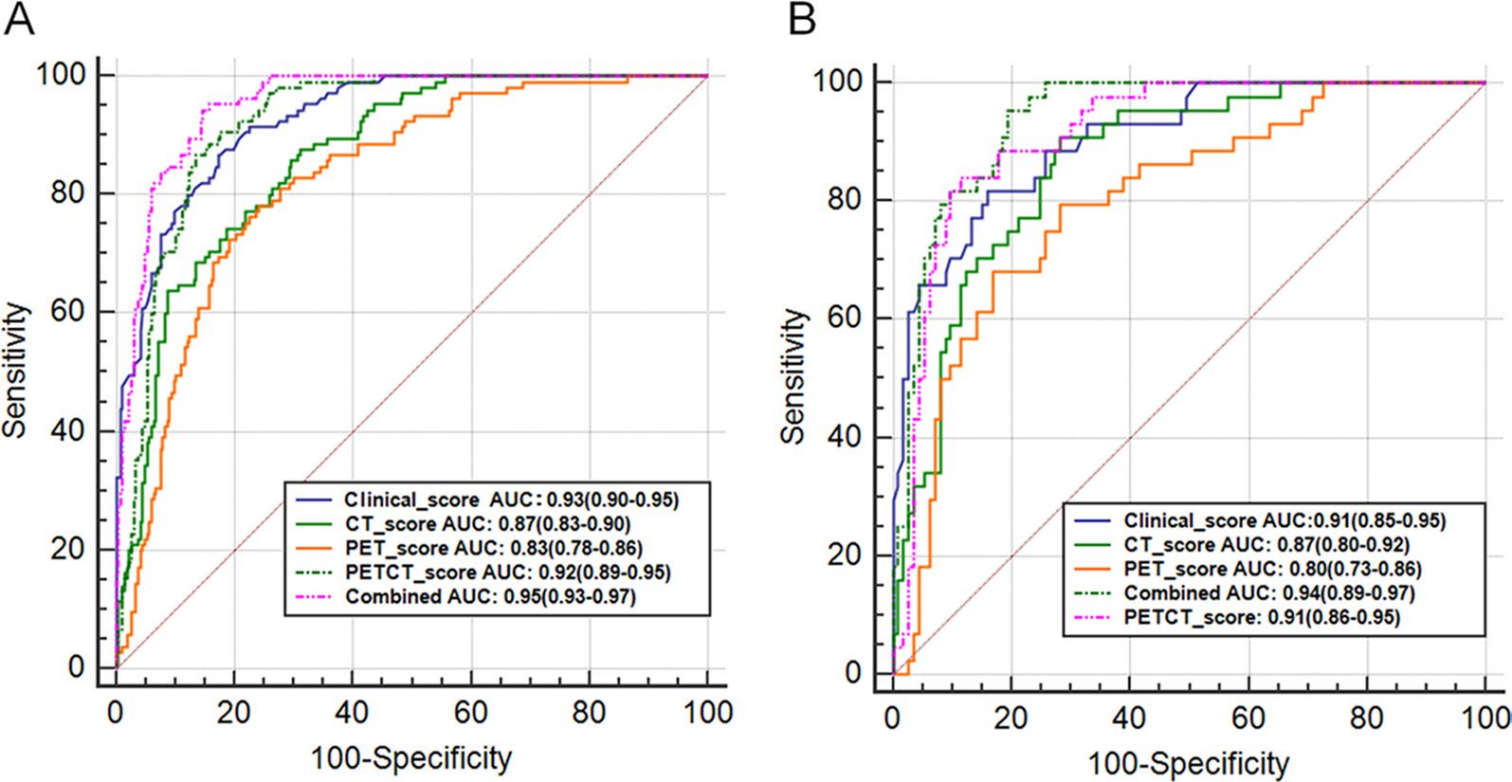
ROC curve analysis of five predictive models, including the clinical model, CT radiomic model, PET radiomic model, PET/CT radiomic model, and combined PET/CT radiomics-clinical model in the training group (**A**) and test group (**B**), respectively

The results showed that all three models (PET/CT, CT, and PET) could predict LNM with cut-off values of -0.66, -0.75, and -0.13, respectively (Additional file 2: Figures S2C, S3C and S4C). The cut-off values were obtained based on Youden's J statistic, and the optimal cut-off was defined as the threshold that maximizes the distance to the identity (diagonal) line. The value can be abbreviated as “y.” The optimum criterion is the max sensitivity + specificity. To determine the stability of the models, 100 rounds of repeated cross-validation were performed (Additional file 1: Figure S2D). The AUC values of the PET/CT, CT and PET radiomics models in the training group were 0.92 (95% CI: 0.89–0.95), 0.87 (95% CI: 0.83–0.90), and 0.83 (95% CI: 0.78–0.86), respectively. The AUC values of these three models in the test group were 0.91 (95% CI: 0.86–0.95), 0.87 (95% CI: 0.80–0.92), and 0.80 (95% CI: 0.73–0.86), respectively (Fig. 2). The sensitivity, specificity, and accuracy of the PET/CT, CT, and PET radiomics models for predicting the thoracic LNM of lung adenocarcinoma in the training and test groups are shown in Table 2. The DeLong test showed that the PET/CT model outperformed the PET and CT models (*p* < 0.05) in the training group (Table 3).

**Table 2:** The performance of 5 different models for prediction of lymph metastasis of lung adenocarcinoma

Models	AUC (95% CI)	ACC (95% CI)	SEN	SPE	PPV	NPV
*Training group*						
PET/CT	0.92 (0.89–0.95)	0.865 (0.826–0.898)	0.865	0.867	0.943	0.717
CT	0.87 (0.83–0.90)	0.741 (0.694–0.785)	0.688	0.876	0.934	0.526
PET	0.83 (0.78–0.86)	0.765 (0.719–0.808)	0.759	0.781	0.898	0.562
Clinical	0.93 (0.90–0.95)	0.838 (0.797–0.974)	0.664	0.94	0.867	0.827
PET/CT + Clinical	0.95 (0.93–0.97)	0.879 (0.841–0.91)	0.717	0.974	0.943	0.853
*Test group*						
PET/CT	0.91 (0.86–0.95)	0.873 (0.81–0.92)	0.885	0.841	0.935	0.74
CT	0.87 (0.80–0.92)	0.771 (0.697–0.834)	0.717	0.909	0.953	0.556
PET	0.80 (0.73–0.86)	0.79 (0.718–0.851)	0.832	0.682	0.87	0.612
Clinical	0.91 (0.85–0.95)	0.783 (0.711–0.845)	0.578	0.925	0.841	0.761
PET/CT + Clinical	0.94 (0.89–0.97)	0.847 (0.781–0.89)	0.656	0.978	0.955	0.805

AUC: area under the curve; CI: confidence interval; ACC: accuracy; SEN: sensitivity; SPE: specificity; PPV: positive predictive value; NPV: negative predictive value. The PET/CT, CT, and PET models represent PET/CT, CT, and PET radiomics models, respectively

**Table 3:** DeLong test of ROC curves between different models

Comparisons	Training		Testing	
	Z score	*p* value	Z score	*p* value
PET/CT vs. PET model	5.157	< 0.001	3.653	< 0.001
PET/CT vs. CT model	3.514	< 0.001	1.931	0.054
PET vs. CT model	1.299	0.194	1.35	0.177
Integrated vs. PET/CT model	3.943	< 0.001	1.65	0.099
Integrated vs. Clinical model	3.257	< 0.001	2.011	0.044
PET/CT vs. Clinical model	0.484	0.628	0.268	0.788

The PET/CT, PET, and CT models represent PET/CT, PET, and CT radiomics models,respectively; integrated model represent PET/CT radiomics-clinical model

### Development of a clinical model for predicting the thoracic LNM of lung adenocarcinoma

Further steps were taken to establish a clinical model for predicting the thoracic LNM of lung adenocarcinoma. After the screening of clinical models using univariate logistic analysis of clinical features, it was found that clinical characteristics, including pleural traction, size of the solid component, and location, were statistically significant in predicting thoracic LNM in the training group (Table 4). According to the results of multivariate logistic regression analysis, size of the solid component and location of the lesion were independent predictors (*p* < 0.05) of the LNM of lung adenocarcinoma, as shown in Table 5. The AUC values of the clinical model in the training and test groups were 0.93 (95% CI: 0.90–0.95) and 0.91 (95% CI: 0.85–0.95), respectively (Table 2).

**Table 4:** Univariate logistic analysis of clinical features and lymph node metastasis

Variables	OR	*p* value
Pleural traction	5.01 (3.05–8.29)	< 0.001
Solid composition	37.99 (18.69–87.63)	< 0.001
Locations	1.18 (1.02–1.36)	0.027

**Table 5:** Multivariate logistic analysis of clinical and radiomic features and lymph node metastasis

Variables	OR	*p* value
Solid composition	13.32 (5.92–33.61)	< 0.001
Locations	1.28 (1.01–1.63)	0.044
Radscore	2.04 (1.55–2.78)	< 0.001
Intercept	0.01 (0–0.04)	< 0.001

### Construction of an integrated PET/CT radiomics-clinical model for predicting the thoracic LNM of lung adenocarcinoma

**Fig. 3:**
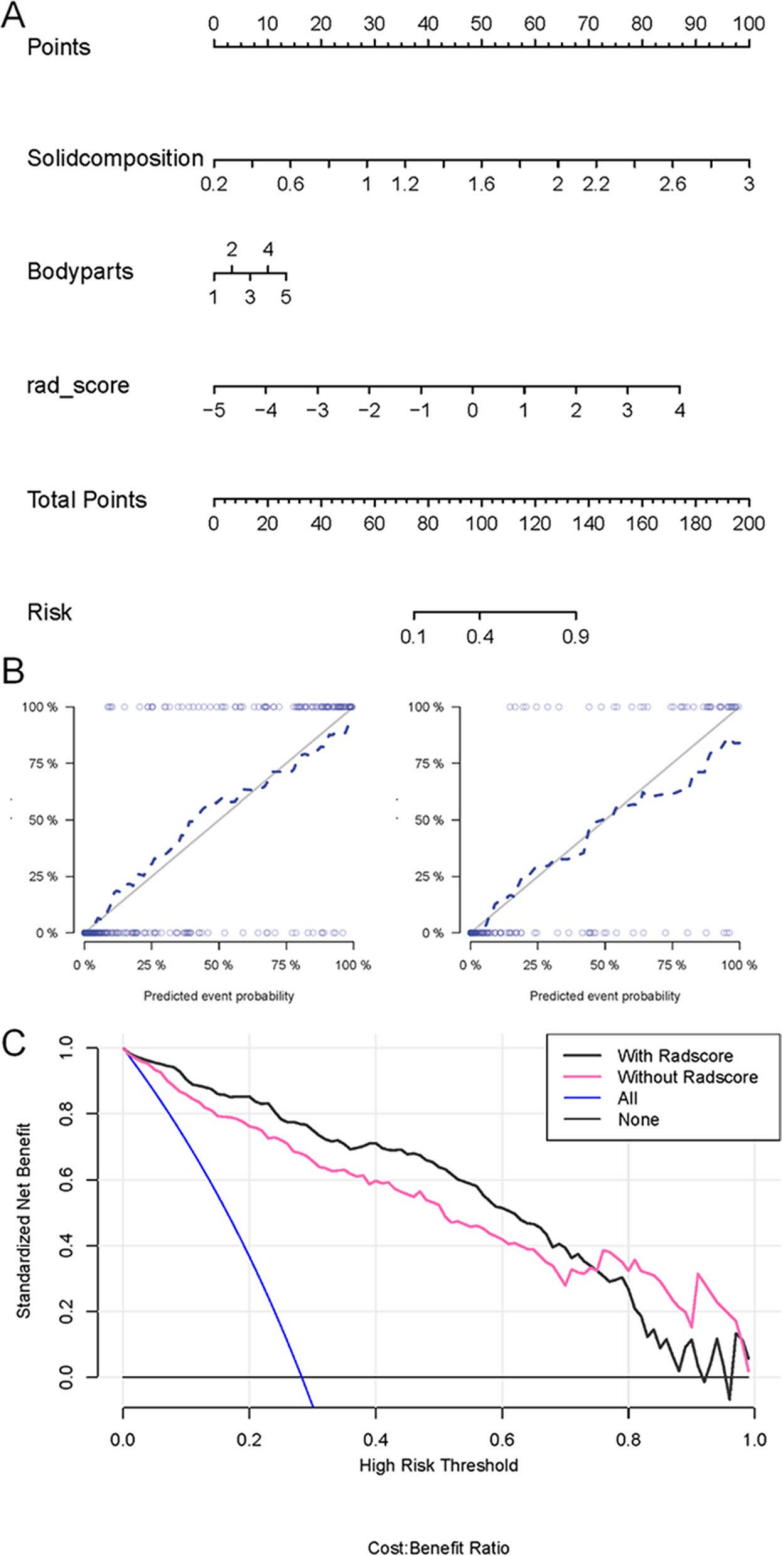
Evaluation of the performance of the integrated PET/CT molecular radiomics-clinical model. **A** The nomogram was developed by combining the PET/CT radiomic score and the clinical features of solid composition and location/body part (1, 2, 3, 4, 5 represent the upper lobe, middle lobe, and lower lobe of the right lung and the upper lobe and lower lobe of the left lung, respectively). **B** Calibration curve with the Hosmer–Lemeshow test of the nomogram in the training cohort (left panel) and test cohort (right panel). The calibration curve shows the calibration of the model in terms of the consistency between the predicted risk of thoracic LNM and the real observed thoracic LNM status. The x-axis represents the predicted risk of thoracic LNM, and the y-axis represents the real thoracic LNM status. **C** Decision curve analysis of the nomograms. The y-axis measures the standardized net benefit. The dark line represents the PET/CT molecular radiomics-clinical nomogram model, the red line represents the clinical features nomogram, the grey line represents the assumption that all patients are negative for thoracic LNM, and the blue line represents the assumption that all patients are positive for thoracic LNM

The integrated PET/CT molecular radiomics-clinical logistic regression model was constructed using the radiomics score and two independent clinical risk factors, and the results are shown in the nomogram in Fig. 3A. In both groups, the PET/CT molecular radiomics-clinical model showed satisfactory performance in predicting LNM (Table 2). The AUC values of the training and test groups were 0.95 (95% CI: 0.93–0.97) and 0.94 (95% CI: 0.89–0.97), respectively. The AUC values of the PET/CT radiomics-clinical, PET/CT radiomics, and clinical models were compared using the DeLong test. It was found that the PET/CT radiomics-clinical model significantly outperformed the PET/CT radiomics model and the clinical model alone (Table 3). The calibration curve of the nomogram, shown in Fig. 3B, displays good calibration of the nomogram. Finally, the clinical usefulness of these models was compared using DCA. When the threshold probability of predicting lung adenocarcinoma LNM is between 1 and 70%, the application of the PET/CT molecular radiomics-clinical model in predicting the thoracic LNM of lung adenocarcinoma has greater advantages than the clinical model (Fig. 3C).

## Discussion

Patients with occult thoracic LNM of lung adenocarcinoma tend to have short disease-free survival and overall survival. Although CT has been routinely used in the diagnosis of LNM of lung cancer, it shows tumour lesions based on only morphological characteristics and thus suffers from significant limitations. In contrast, PET/CT provides a high accuracy rate in diagnosing local LNM, as it relies on the metabolic characteristics of a tissue, and metabolic changes often occur ahead of morphologic changes [5]. Upon the intake of an imaging reagent, metastatic lesions of lung adenocarcinoma can be judged from the perspective of molecular metabolism [5, 29]. Importantly, PET/CT radiomics has been investigated in lung cancers [30–32]. For example, Mu et al. reported that deep learning of PET/CT images could predict PD-L1 status and immunotherapy response in NSCLC [33]. Du et al. reported that a PET/CT radiomics nomogram showed potential for the individualized differential diagnosis of solid active pulmonary tuberculosis and lung cancer [34]. In our previous studies [23, 24], a PET/CT radiomics model was developed to predict epidermal growth factor receptor (EGFR) mutation and anaplastic lymphoma kinase (ALK) rearrangement status in lung adenocarcinoma. This study aimed to predict the thoracic LNM of lung adenocarcinoma by PET/CT radiomics.

Multiple studies have demonstrated the value of CT radiomics in predicting the LNM of lung cancer [16, 17, 35]. For example, Yang et al. developed a nomogram with 14 CT radiomics features to predict LNM in solid lung adenocarcinoma, and the results showed that the AUC values for the training and validation cohorts were 0.871 and 0.856, respectively [16]. Moreover, the LASSO algorithm was used to choose the best set of CT radiomics features and develop a predictive LNM model in IA NSCLC patients based on radiomics and clinical features. The predictive performance for LNM of the combined model was further improved (the AUC values for training and testing were 0.911 and 0.860, respectively) [17]. In addition, PET/CT has also been used to predict LNM in NSCLC [8, 9]. However, only one PET/CT parameter was used alone to predict the LNM of NSCLC in these studies, which yielded high specificity but low sensitivity of diagnosis [8, 9]. In this study, 10 parameters of PET/CT images were extracted using the LASSO algorithm and were used to construct a predictive radiomics model, thus showing more sensitivity in predicting the thoracic LNM of lung adenocarcinoma. The AUC value of the ROC curve of the radiomics model was 0.92 in the training group and 0.91 in the test group. In addition, the clinical model (the visual assessment) was constructed by the subjective sign of the solid component and location of lung adenocarcinoma after feature selection, and the AUC value reached 0.93 in the training group and 0.91 in the test group. It was found that both the clinical model and radiomics model achieved similarly good performance outcomes in predicting LNM. Furthermore, compared with the PET/CT clinical model, the integrated PET/CT molecular radiomics-clinical model shows more power for predicting the local LNM of lung adenocarcinoma. The AUC value increased from 0.93 to 0.95 in the training group and from 0.91 to 0.94 in the test group.

The present results are significant in several major aspects as follows. First, the location of lung cancer is closely related to LNM [36, 37]. Ketchedjian et al*.* showed that the incidence of LNM increased as the size of peripheral T1 tumours of lung adenocarcinoma increased, whereas central T1 tumours demonstrated a 50% incidence of lymph node involvement irrespective of tumour size [36]. This study found that the location of lung adenocarcinoma was an important factor in predicting the LNM of lung adenocarcinoma. Second, it has been reported that there is a strong correlation between the diameter of solid components on CT images and the invasive components revealed by pathology, and the size of the solid component is an important factor affecting prognosis [19, 38, 39]. In this study, the solid component size was shown to serve as an effective predictor of thoracic LNM. Third, the nomogram provides a quantitative and intuitive method for clinicians to predict the LNM of lung adenocarcinoma. Finally, our study addresses the limitation of the CT radiomics model where the LNM of lung adenocarcinoma is predicted from CT morphology alone, while incorporating PET radiomics to indicate the level of tumour molecular metabolism. The overall objective of the current study is to further confirm the application value of PET radiomics. To date, there is no literature report comparing PET/CT and CT radiomics models in the prediction of the LNM of lung adenocarcinoma. This study found that PET/CT radiomics was superior to CT radiomics in the training group, indicating that PET radiomics has certain diagnostic value in predicting the LNM of lung adenocarcinoma.

Despite the promising statistical results, several limitations of this study need to be acknowledged. First, in view of the single-centre retrospective nature of the current study, a multicentre study with a larger sample size should be conducted for further verification. Second, it is time-consuming for radiologists to semi-manually delineate the segmentation of lung adenocarcinoma lesions by means of ITK software. It is expected that with the development of artificial intelligence software such as deep learning, fully automatic computer segmentation can be realized [20, 40, 41]. Third, this study lacks external validation to refine the proposed model. Finally, due to limited time after surgery and incomplete follow-ups, a predictive model for survival rate has yet to be established.

## Conclusion

In summary, the PET/CT molecular radiomics-clinical model demonstrated its high diagnostic value in predicting the thoracic LNM of lung adenocarcinoma. A PET/CT molecular radiomics-clinical nomogram model was developed as a visualization tool to help predict thoracic LNM in newly diagnosed lung adenocarcinoma patients.

## Supplementary Information

**Additional file 1. Figure S1**: Flowchart of lung adenocarcinoma patient selection.

**Additional file 2. Figure S2**: Construction of a PET/CT radiomics model based on PET/CT images. (**A**) A total of 10 radiomics features were identified by mRMR and LASSO logistic regression based on PET and CT features. (**B**) List of 10 radiomics features chosen to construct the PET/CT radiomics model. (**C**) Representative results of the PET/CT radiomics model for predicting thoracic LNM in the training (left) and test (right) groups of lung adenocarcinoma patients. 0, negative thoracic LNM; 1, positive thoracic LNM. (**D**) Cross-validation analysis showed that the PET/CT radiomics model has good reliability for predicting thoracic LNM in the training (left) and test (right) groups of lung adenocarcinoma patients

**Additional file 3. Figure S3**: Construction of a CT radiomics model based on CT images. (**A**) A total of 12 radiomics features were identified by mRMR and LASSO logistic regression based on CT features. (**B**) List of 12 radiomics features chosen to construct the CT radiomics model. (**C**) Representative results of the CT radiomics model for predicting thoracic LNM in the training (left) and test (right) groups of lung adenocarcinoma patients. 0, negative thoracic LNM; 1, positive thoracic LNM

**Additional file 4. Figure S4**: Construction of a PET radiomics model based on PET images. (**A**) A total of 10 radiomic features were identified by mRMR and LASSO logistic regression based on PET features. (**B**) List of 10 radiomisc features chosen to construct the PET radiomics model. (**C**) Representative results of the PET radiomics model for predicting thoracic LNM in the training (left) and test (right) groups of lung adenocarcinoma patients. 0, negative thoracic LNM; 1, positive thoracic LNM

**Additional file 5. Table S1**: The intra-and interobserver ICCs of the consistency of lesion segmentation

**Additional file 6. Methods**: The formula for the calculation of PET/CT, CT, PET radiomics scores

## Data Availability

The datasets used and/or analysed during the current study are available from the corresponding author on reasonable request.

## Abbreviations

LNM: Lymph node metastasis
NSCLC: Non-small-cell lung cancer
DCA: Decision curve analysis
PET/CT: Positron emission tomography/computed tomography
SUVmax: Maximum standardized uptake value
CEA: Carcinoembryonic antigen
VOI: Volume of interest
ICCs: Inter- and intraclass correlation coefficients
GLCM: Grey level co-occurrence matrix
RLM: Run length matrix
GLSZM: Grey level size zone matrix
mRMR: Maximum relevance minimum redundancy
LASSO: Least absolute shrinkage and selection operator
AUC: Area under the curve
VIF: Variance inflation factor
SMOTE: Synthetic minority oversampling technique
EGFR: Epidermal growth factor receptor
ALK: Anaplastic lymphoma kinase
