# Supplementary figures and images for: Development of a PET/CT molecular radiomics-clinical model to predict thoracic lymph node metastasis of invasive lung adenocarcinoma ≤ 3 cm in diameter
Source: EJNMMI Res. 2022 Apr 21;12:23. doi: 10.1186/s13550-022-00895-x (PMC9023644; doi:10.1186/s13550-022-00895-x)

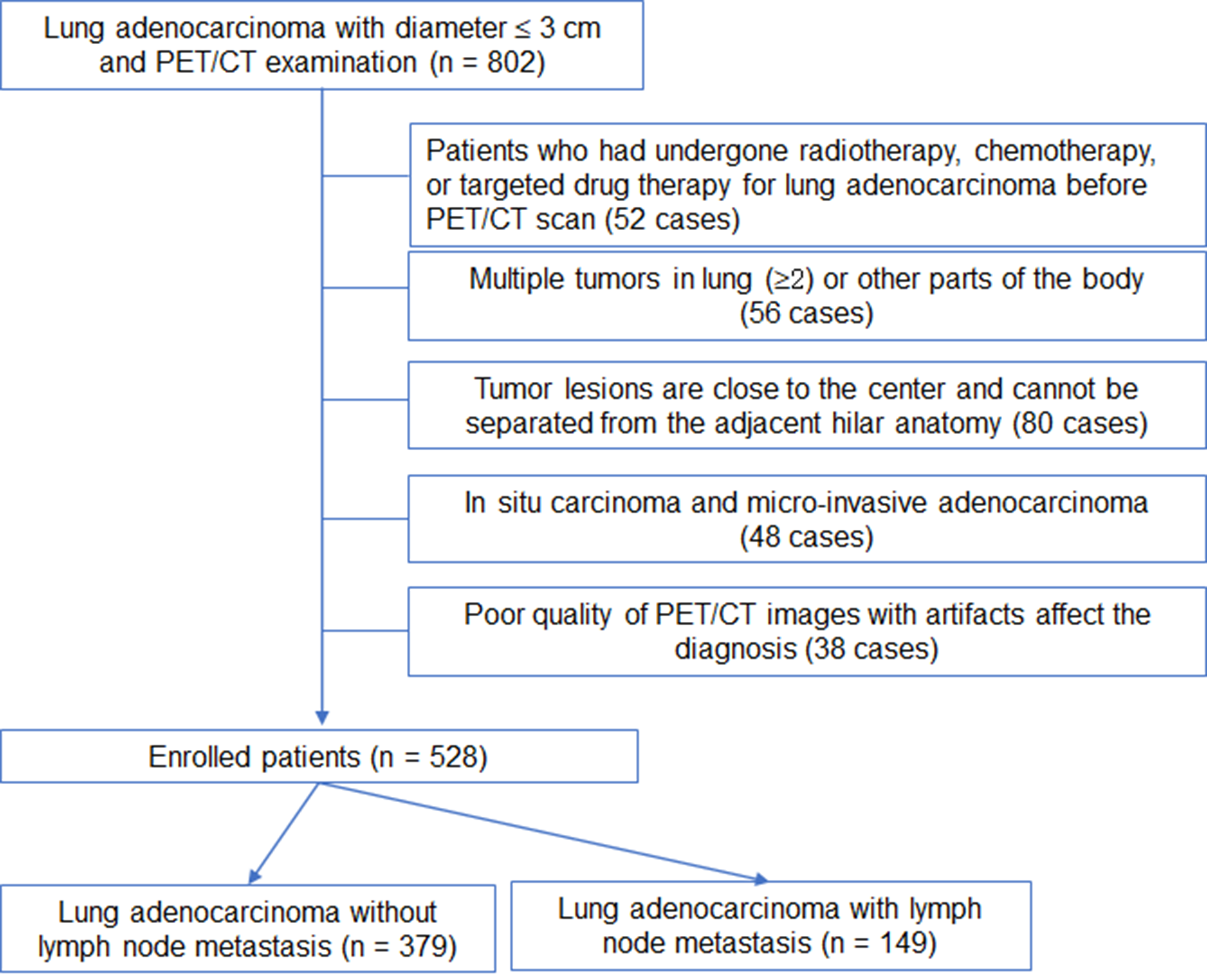

Supplement: Supplementary file 1 — Additional file 1. Figure S1: Flowchart of lung adenocarcinoma patient selection. [file 13550_2022_895_MOESM1_ESM.tif]

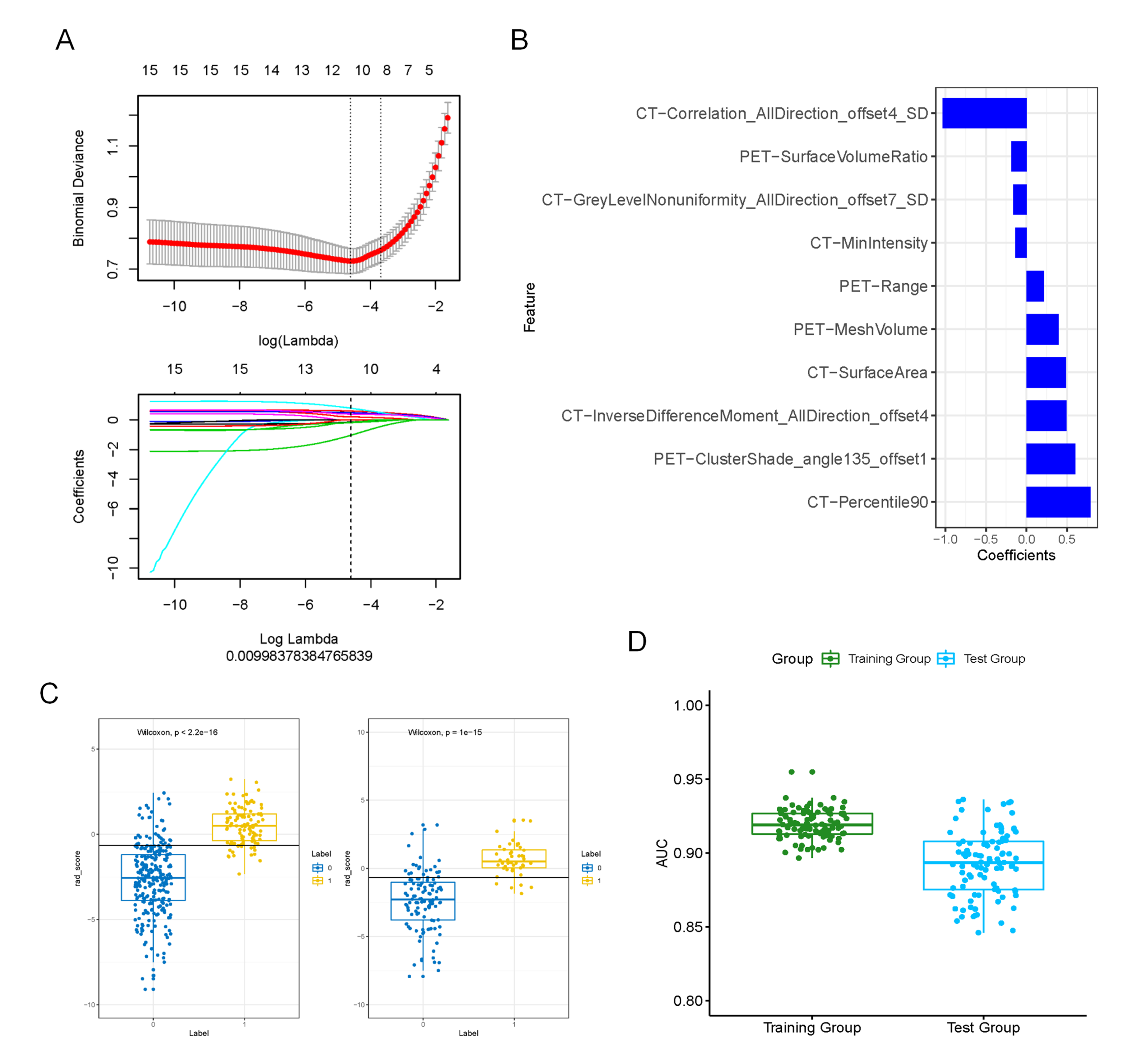

Supplement: Supplementary file 2 — Additional file 2. Figure S2: Construction of a PET/CT radiomics model based on PET/CT images. (A) A total of 10 radiomics features were identified by mRMR and LASSO logistic regression based on PET and CT features. (B) List of 10 radiomics features chosen to construct the PET/CT radiomics model. (C) Representative results of the PET/CT radiomics model for predicting thoracic LNM in the training (left) and test (right) groups of lung adenocarcinoma patients. 0, negative thoracic LNM; 1, positive thoracic LNM. (D) Cross-validation analysis showed that the PET/CT radiomics model has good reliability for predicting thoracic LNM in the training (left) and test (right) groups of lung adenocarcinoma patients [file 13550_2022_895_MOESM2_ESM.tif]

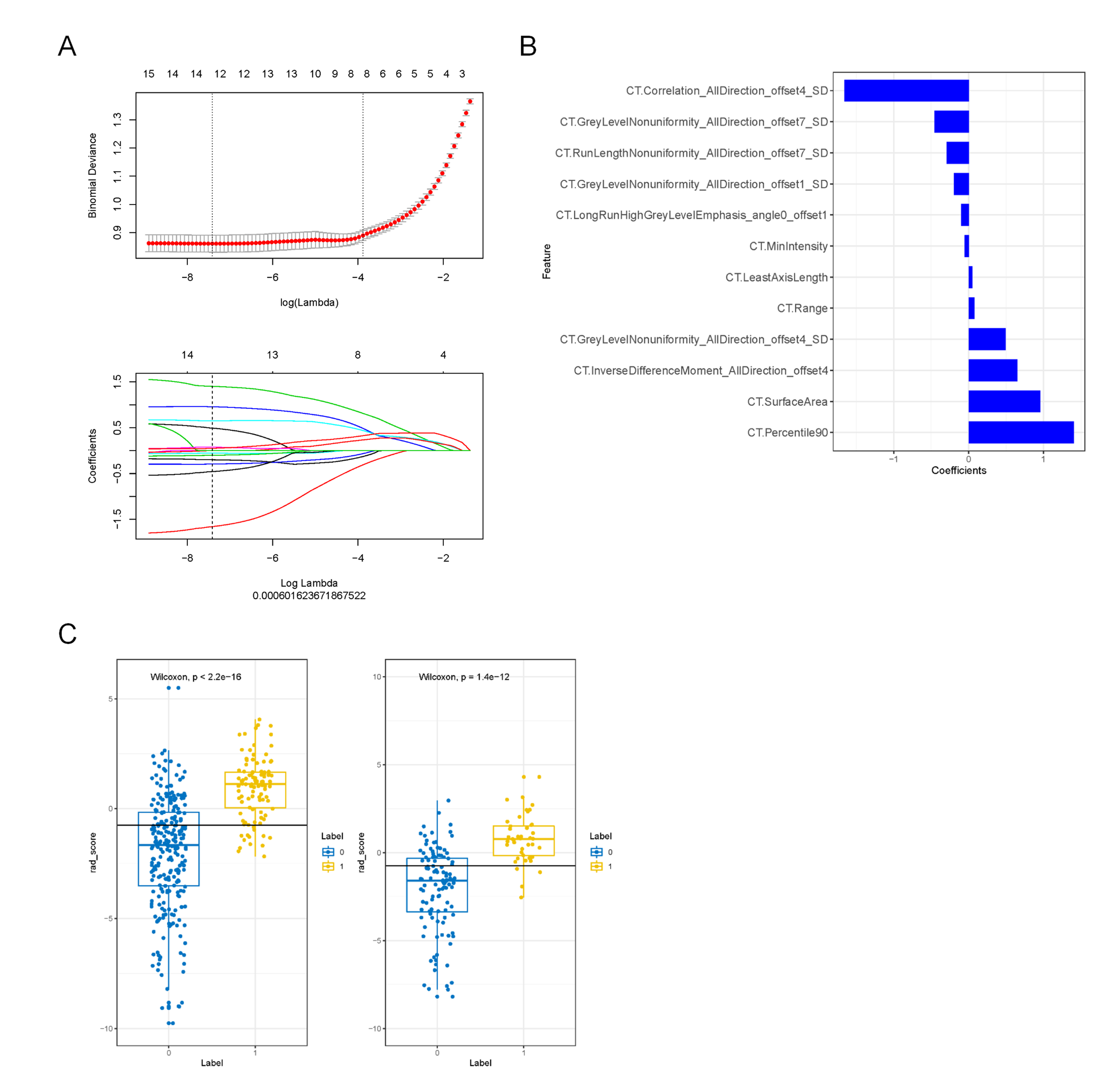

Supplement: Supplementary file 3 — Additional file 3. Figure S3: Construction of a CT radiomics model based on CT images. (A) A total of 12 radiomics features were identified by mRMR and LASSO logistic regression based on CT features. (B) List of 12 radiomics features chosen to construct the CT radiomics model. (C) Representative results of the CT radiomics model for predicting thoracic LNM in the training (left) and test (right) groups of lung adenocarcinoma patients. 0, negative thoracic LNM; 1, positive thoracic LNM [file 13550_2022_895_MOESM3_ESM.tif]

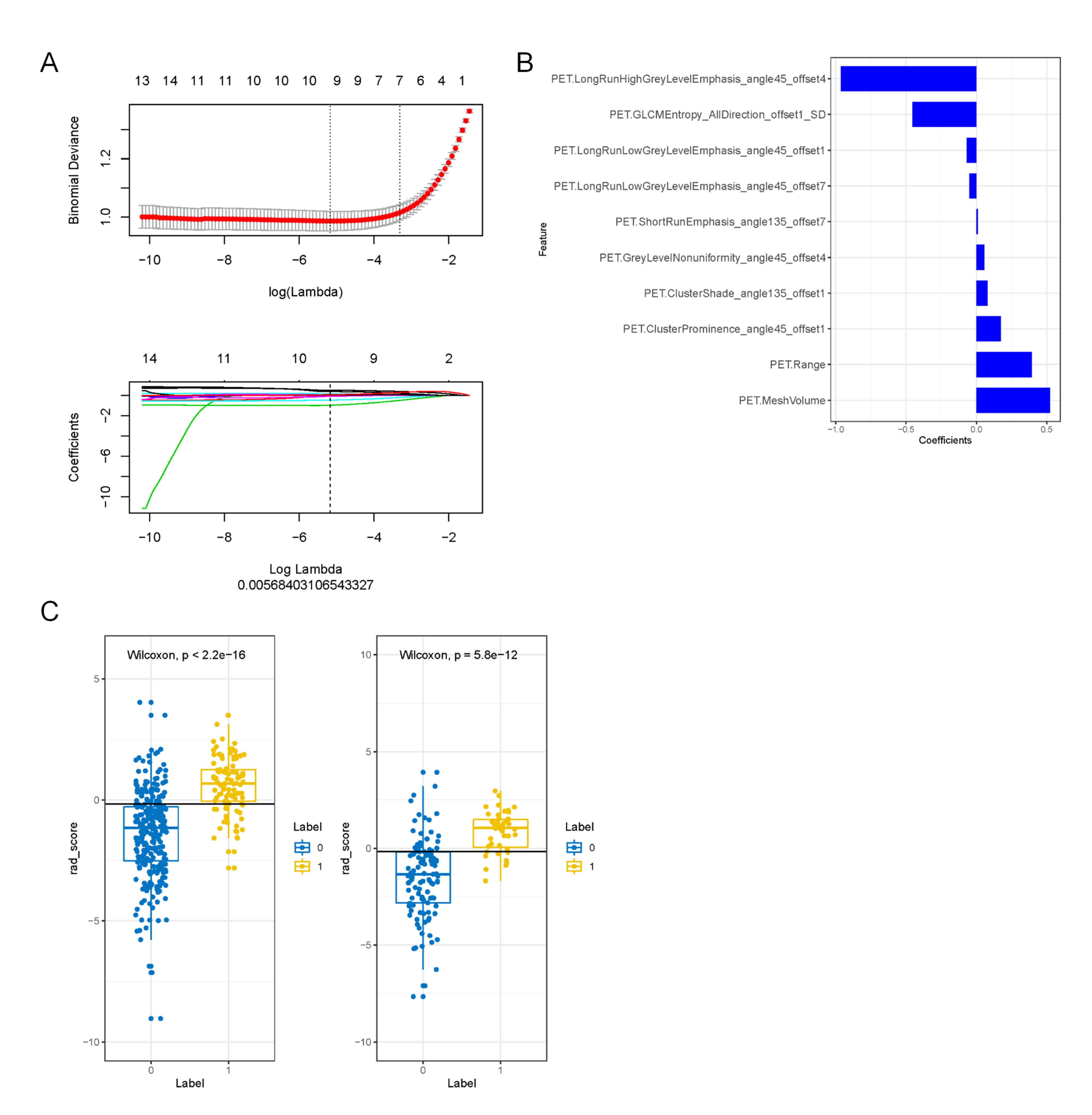

Supplement: Supplementary file 4 — Additional file 4. Figure S4: Construction of a PET radiomics model based on PET images. (A) A total of 10 radiomic features were identified by mRMR and LASSO logistic regression based on PET features. (B) List of 10 radiomisc features chosen to construct the PET radiomics model. (C) Representative results of the PET radiomics model for predicting thoracic LNM in the training (left) and test (right) groups of lung adenocarcinoma patients. 0, negative thoracic LNM; 1, positive thoracic LNM [file 13550_2022_895_MOESM4_ESM.tif]
