# Supplementary material for: Development of a PET/CT molecular radiomics-clinical model to predict thoracic lymph node metastasis of invasive lung adenocarcinoma ≤ 3 cm in diameter
Source: EJNMMI Res. 2022 Apr 21;12:23. doi: 10.1186/s13550-022-00895-x (PMC9023644; doi:10.1186/s13550-022-00895-x)
Supplement: Supplementary file 5 — Additional file 5. Table S1: The intra-and interobserver ICCs of the consistency of lesion segmentation [file 13550_2022_895_MOESM5_ESM.docx]

**Supplementary Table**

**Supplementary Table S1.** The intra-and inter-observer ICCs of the consistency

of lesions segmentation.

|  | CT Images | PET Images |
| --- | --- | --- |
| Intra-group ICC | 0.06~1 | 0.32~1 |
| Remains (ICC>0.75) | 191 | 271 |
| Inter-group ICC | 0.15~1 | 0.3~1 |
| Remains (ICC>0.75) | 202 | 256 |
