# Supplementary material for: Development of a PET/CT molecular radiomics-clinical model to predict thoracic lymph node metastasis of invasive lung adenocarcinoma ≤ 3 cm in diameter
Source: EJNMMI Res. 2022 Apr 21;12:23. doi: 10.1186/s13550-022-00895-x (PMC9023644; doi:10.1186/s13550-022-00895-x)
Supplement: Supplementary file 6 — Additional file 6. Methods: The formula for the calculation of PET/CT, CT, PET radiomics scores [file 13550_2022_895_MOESM6_ESM.docx]

**Supplementary Methods**

Radiomics score (radscore) was calculated by summing the selected features weighted by their coefficients. The formula for the calculation of radiomics scores was as follows:

**The formula for PET/CT radiomics score is:**

PET/CT Radscores = 0.493*CT-InverseDifferenceMoment_AllDirection_offset4+0.211*PET-Range+-1.033*CT-Correlation_AllDirection_offset4_SD+-0.139*CT-MinIntensity+0.603*PET-ClusterShade_angle135_offset1+-0.185*PET-SurfaceVolumeRatio+0.488*CT-SurfaceArea+0.79*CT-Percentile90+0.394*PET-MeshVolume+-0.165*CT-GreyLevelNonuniformity_AllDirection_offset7_SD + -1.825

**The formula for CT radiomics score is:**

CT Radscores = 0.492*CT.GreyLevelNonuniformity_AllDirection_offset4_SD+-1.659*CT.Correlation_AllDirection_offset4_SD+1.403*CT.Percentile90+0.958*CT.SurfaceArea+0.65*CT.InverseDifferenceMoment_AllDirection_offset4+-0.199*CT.GreyLevelNonuniformity_AllDirection_offset1_SD+-0.293*CT.RunLengthNonuniformity_AllDirection_offset7_SD+-0.054*CT.MinIntensity+0.077*CT.Range+-0.458*CT.GreyLevelNonuniformity_AllDirection_offset7_SD+0.05*CT.LeastAxisLength+-0.102*CT.LongRunHighGreyLevelEmphasis_angle0_offset1 + -1.454

**The formula for PET radiomics score is:**

PET Radscores = 0.065*PET.ClusterProminence_angle45_offset1+-38.822*PET.Correlation_AllDirection_offset1_SD+0.457*PET.Variance+1.014*PET.GreyLevelNonuniformity_angle0_offset1+-1.521*PET.SurfaceVolumeRatio+-0.426*PET.VoxelValueSum+-0.584*PET.GLCMEntropy_AllDirection_offset1_SD+0.015*PET.ClusterProminence_AllDirection_offset1_SD+0.296*PET.MeshVolume+-0.334*PET.LongRunLowGreyLevelEmphasis_angle45_offset1+0.752*PET.GLCMEnergy_AllDirection_offset1_SD+0.309*PET.ClusterShade_angle135_offset1+-1.062*PET.GreyLevelNonuniformity_angle90_offset4 + -5.083
